# Implications of mitochondrial dynamics on neurodegeneration and on hypothalamic dysfunction

**DOI:** 10.3389/fnagi.2015.00101

**Published:** 2015-06-10

**Authors:** Antonio Zorzano, Marc Claret

**Affiliations:** ^1^Molecular Medicine Program, Institute of Research in Biomedicine (IRB Barcelona)Barcelona, Spain; ^2^Departament de Bioquímica i Biologia Molecular, Facultat de Biologia, Universitat de BarcelonaBarcelona, Spain; ^3^CIBER de Diabetes y Enfermedades Metabólicas Asociadas, Instituto de Salud Carlos IIIBarcelona, Spain; ^4^Diabetes and Obesity Research Laboratory, Institut d'Investigacions Biomèdiques August Pi i SunyerBarcelona, Spain

**Keywords:** mitochondrial fission, mitochondrial fusion, mitofusin 2, obesity, food intake, neurodegenerative diseases

## Abstract

Mitochondrial dynamics is a term that encompasses the movement of mitochondria along the cytoskeleton, regulation of their architecture, and connectivity mediated by tethering and fusion/fission. The importance of these events in cell physiology and pathology has been partially unraveled with the identification of the genes responsible for the catalysis of mitochondrial fusion and fission. Mutations in two mitochondrial fusion genes (*MFN2* and *OPA1*) cause neurodegenerative diseases, namely Charcot-Marie Tooth type 2A and autosomal dominant optic atrophy (ADOA). Alterations in mitochondrial dynamics may be involved in the pathophysiology of prevalent neurodegenerative conditions. Moreover, impairment of the activity of mitochondrial fusion proteins dysregulates the function of hypothalamic neurons, leading to alterations in food intake and in energy homeostasis. Here we review selected findings in the field of mitochondrial dynamics and their relevance for neurodegeneration and hypothalamic dysfunction.

## Introduction

Mitochondrial filaments were first reported in the mid-1800s upon the discovery of mitochondria. However, it was not until the 1990s that the improvement of mitochondrial dyes permitted the visualization of mitochondrial motion in different cellular models (Bereiter-Hahn and Voth, 1994; Nunnari et al., 1997; Cortese et al., 1998). Mitochondrial dynamics alludes to the movement of mitochondria along the cytoskeleton and also to changes in mitochondrial morphology, both parameters being controlled by fusion and fission events, which in turn lead to these organelles forming tubular or branched reticular networks.

The discovery of the first gene that participates in mitochondria fusion in *Drosophila melanogaster* came about in 1997 (Hales and Fuller, 1997). In the last years, genes that participate in mitochondrial fusion or fission have been identified (Yaffe, 1999). Although considerable advances have been made in the field of mitochondrial dynamics in recent years, the molecular elements that regulate fusion and fission remain unclear. In this regard, the physiological relevance of mitochondrial dynamics in mammals is not precisely understood, and the proteins that determine differences in mitochondrial morphology among distinct cell types need to be clarified.

Mitochondrial dynamics is required to facilitate inter-mitochondrial complementation, and it also participates in ensuring that mitochondria undergo maximal hyperfusion under conditions of cellular stress (Chen and Chan, 2005; Tondera et al., 2009). The fragmentation of mitochondria is also crucial in order for these organelles to undergo mitophagy (Twig et al., 2008), and balanced mitochondrial dynamics is key to the maintenance of an appropriate cell metabolism (Bach et al., 2003; Sebastian et al., 2012).

## Major processes and proteins involved in mitochondrial dynamics

Here we will review the proteins that participate in mitochondrial fusion and mitochondrial fission.

### The mitochondrial fusion machinery

Mitochondrial compartmentalization is ensured by fusion of the inner and outer mitochondrial membranes (Meeusen et al., 2004). The main proteins involved in this process are the outer membrane GTPases Mitofusins (Mfn1 and Mfn2) (Chen et al., 2003; Ishihara et al., 2004) and the inner membrane GTPase Optic atrophy 1 (OPA1) (Cipolat et al., 2004; Ishihara et al., 2006).

Mfn1 and Mfn2 are integral outer mitochondrial membrane proteins and modulate mitochondrial morphology by promoting mitochondria tethering and fusion (Koshiba et al., 2004). The functions of Mfn1 and Mfn2 seems to overlap, since Mfn1 partially rescues the defects caused by Mfn2 mutation (Detmer and Chan, 2007). *Mfn1* and *Mfn2* genes are widely expressed. Mfn1 gene expression is high in heart and is expressed at lower level in other human tissues. Mfn2 transcripts are abundant in heart and skeletal muscle and present at lower levels in other tissues (Santel et al., 2003).

Mfn1 shows two transmembrane domains at the C-terminus of the protein, near a heptad-repeat (HR) domain (Santel et al., 2003). The N-terminal region of Mfn1 contains a GTP-binding domain followed by a heptad-repeat domain (HR1) (Santel et al., 2003; Koshiba et al., 2004). The C-terminal HR domain is considered to mediate the first step of mitochondrial fusion, which consists of the tethering of two adjacent mitochondria through the formation of a dimeric antiparallel coiled-coil structure (Koshiba et al., 2004). These dimeric structures can be homotypic (Mfn1-Mfn1 or Mfn2-Mfn2) or heterotypic (Mfn1-Mfn2) (Chen et al., 2003; Koshiba et al., 2004). In addition, Mfn1 shows higher GTPase activity than Mfn2 (Ishihara et al., 2004). In this respect, mitochondria containing Mfn1 show greater tethering efficiency than mitochondria with Mfn2 (Ishihara et al., 2004).

Mfn1 shows both transcriptional and post-transcriptional or post-translational regulation (Santel et al., 2003). In this regard, Mfn1 is regulated by PGC-1α during postnatal cardiac growth (Martin et al., 2014). In contrast, Mfn1 is repressed by dexamethasome in liver and in hepatoma cells (Hernandez-Alvarez et al., 2013) and by microRNA 140 in cardiomyocytes (Li et al., 2014). Regarding post-translational regulation, Mfn1 undergoes ubiquitination mediated by MARCH-V (also named MITOL) or Parkin (Gegg et al., 2010; Park et al., 2010; Tanaka et al., 2010; Park and Cho, 2012), and deacetylation and activation mediated by HDAC6 (Lee et al., 2014). Mfn1 may be regulated through binding to MIB, a member of the quinone oxidoreductase subfamily of zinc-containing alcohol dehydrogenase proteins (Eura et al., 2006), and overexpression of MIB induces mitochondrial fragmentation, whereas MIB knockdown causes enhanced mitochondrial network structures (Eura et al., 2006).

As mentioned, Mfn2 is an integral outer mitochondrial membrane protein, which exposes both terminal ends to the cytosol (Rojo et al., 2002). The N-terminal GTPase activity of Mfn2 is key for its function in mitochondrial fusion (Chen et al., 2003; Eura et al., 2003). Mfn2 is essential for embryonic development, and ablation of Mfn2 causes placental dysfunction (Chen et al., 2003). Mfn2 exerts a key role in brain, and protects against neurodegeneration in the cerebellum (Chen et al., 2007) as well as in dopaminergic neurons (Lee et al., 2012; Pham et al., 2012). In addition, Mfn2 repression in neurons leads to a delayed cell death upon excitotoxicity (Martorell-Riera et al., 2014). Mfn2 has been proposed to regulate cell proliferation and mitochondrial metabolism (Bach et al., 2003; Chen et al., 2004a, 2014; Pich et al., 2005). In addition, this protein promotes insulin signaling, and deficiency in muscle or liver causes impaired insulin signaling caused by excessive JNK activity and phosphorylation of IRS proteins at serine residues—the latter inhibiting the capacity to activate PI-3 kinase and downstream elements of the pathway (Sebastian et al., 2012). Whether Mfn2 modulates insulin signaling in other tissues such as brain, remains unknown.

The transcription factor Sp1 binds to and drives Mfn2 gene transcription both in skeletal muscle and in smooth muscle cells (Sorianello et al., 2012). The transcription factor Estrogen-Related Receptor-alpha (ERRα) also binds to the human Mfn2 promoter and stimulates transcription (Soriano et al., 2006). The transcription of Mfn2 is stimulated by the coactivators PGC-1α and PGC-1β —key factors in mitochondrial biogenesis (Scarpulla et al., 2012)— through physical/functional interaction with ERRα (Soriano et al., 2006; Liesa et al., 2008). Thus, PGC-1α or PGC1β overexpression induces Mfn2 in cells (Soriano et al., 2006; Liesa et al., 2008). PGC-1β is also necessary for the maintenance of Mfn2 in tissues, and PGC-1β KO mice show reduced Mfn2 in metabolically-relevant tissues (Liesa et al., 2008). In keeping with this, PGC-1β influences mitochondrial morphology through the promotion of mitochondrial elongation, and enhanced mitochondrial fusion (Liesa et al., 2008). Interestingly. overexpression of PGC-1β causes mitochondrial elongation in wild-type, and in Mfn1 KO MEF cells. However, such overexpression does not cause mitochondrial elongation in Mfn2 KO MEF cells (Liesa et al., 2008), which suggests that Mfn2 is required for the effects of PGC-1β on mitochondrial morphology. These data are also consistent with the reduced mitochondrial volume, independent of changes in mitochondrial number, detected in muscle from PGC-1β KO mice (Lelliott et al., 2006; Liesa et al., 2008). Based on all those evidences, Mfn2 undergoes transcriptional regulation.

In addition to this mechanism of control, Mfn2 is regulateded by proteasomal degradation. In this respect, Mfn2 undergoes ubiquitination catalyzed by Parkin (Gegg et al., 2010; Poole et al., 2010; Tanaka et al., 2010; Ziviani et al., 2010). Mfn2 is also ubiquitinated by the E3 ubiquitin ligases HUWE1, regulated by phosphorylation of JNK, and by the ubiquitin ligase Mul1 (Leboucher et al., 2012; Lokireddy et al., 2012). The processes of Mfn2 ubiquitination lead to Mfn2 degradation. The ubiquitin ligase MARCH-V (or MITOL) catalyzes lysine-63-linked polyubiquitination of Mfn2 but does not drive its proteasomal degradation (Sugiura et al., 2013). Mfn2 levels are also regulated by activation of the deubiquitinase USP30 (Yue et al., 2014).

Optic atrophy gene 1 (OPA1) is also a dynamin-related protein with GTPase activity. This protein is located in the intermembrane space, in the inner mitochondrial membrane and in the cristae volume (Misaka et al., 2002; Olichon et al., 2002; Satoh et al., 2003). OPA1 is essential for mitochondrial fusion (Cipolat et al., 2004; Chen et al., 2005). OPA1 shows high expression in brain, retina, liver, heart, skeletal muscle, and testis (Delettre et al., 2001; Cipolat et al., 2004). OPA1 maintains the normal structure of the optic nerve (Davies et al., 2007), and its deficiency causes alterations in the dendritic morphology of retinal ganglion cells (Williams et al., 2010). In addition, OPA1 promotes neuronal survival following excitotoxicity (Jahani-Asl et al., 2011).

Various OPA1 isoforms have been identified (Olichon et al., 2003; Cipolat et al., 2004; Griparic et al., 2004; Ishihara et al., 2006). The existence of multiple OPA1 isoforms and cleavage mechanisms may explain the role of this protein beyond mitochondrial inner membrane fusion, such as in cristae remodeling, supercomplex formation, and in the regulation of the selective fusion that determines mitochondrial autophagy (Frezza et al., 2006; Twig et al., 2008; Cogliati et al., 2013). OPA1 isoforms are generated from a single gene through alternative splicing and proteolysis. OPA1 isoforms are named as long and short on the basis of their electrophoretic mobility (Delettre et al., 2001; Olichon et al., 2007; Akepati et al., 2008). Both short and long forms are required for mitochondrial fusion, and thus constitutive OPA1 cleavage is required for efficient mitochondrial fusion (Song et al., 2007; DeVay et al., 2009). Some long OPA1 isoforms are constitutively cleaved by the intermembrane space AAA protease YME1L (Griparic et al., 2007; Song et al., 2007). The cleavage of OPA1 is also induced by mitochondrial depolarization, which reduces the abundance of long isoforms, increases the short OPA1 isoforms, and reduces mitochondrial fusion. This inducible OPA1 cleavage is also activated by ATP deficiency and by apoptosis (Baricault et al., 2007; Griparic et al., 2007) and it is catalyzed by the zinc metalloprotease OMA1 (Ehses et al., 2009; Head et al., 2009; Quiros et al., 2012). In this respect, OMA1 ablation causes a deficient mitochondrial activity and function in brown adipose tissue, and obesity (Quiros et al., 2012).

### The mitochondrial fission machinery

Mitochondrial fission describes the fragmentation of a mitochondrion into two. This process is necessary to drive damaged mitochondria through mitophagy (Kim et al., 2007), to pass mitochondria to daughter cells during mitosis, and to regulate apoptosis (Lee et al., 2004). Alterations in fission machinery increase the generation of reactive oxygen species (ROS) and lead to a heterogeneous population of mitochondria with non-uniform mitochondrial DNA distribution (Parone et al., 2008).

Dynamin-related protein 1 (Drp1) plays a key role in the catalysis of mitochondrial fission. Drp1 is a cytosolic protein that is recruited to the outer mitochondrial membrane, where it catalyzes mitochondrial division. Drp1 interacts with the mitochondrial proteins fission protein 1 homolog (Fis1), the mitochondrial fission factor (Mff), MiD49, and MiD51.

Drp1 is found mainly in the cytosol and, as a member of the dynamin protein family, it contains a GTPase domain and a GTPase effector domain. Drp1 assembles into multimeric ring complexes at mitochondrial fission sites, which leads to constriction following GTP hydrolysis to promote division (Bleazard et al., 1999; Van Der Bliek, 1999; Legesse-Miller et al., 2003; Ingerman et al., 2005; Lackner et al., 2009; Mears et al., 2011). Given that Drp 1 lacks domains involved in membrane binding, the recruitment of this molecule at the mitochondrial outer membrane requires the involvement of membrane proteins that act as receptors (Parone et al., 2008).

Drp1 is regulated by post-translational modifications such as S-nitrosylation, sumoylation, ubiquitination, and phosphorylation of serine residues, and they may regulate its recruitment to the mitochondria. S-nitrosylation enhances the pro-fission activity of Drp1 by inducing dimerization and enhancing GTPase activity (Cho et al., 2009). Drp1 S-nitrosylation and mitochondrial fragmentation have been detected by the ß-amyloid protein (Aβ), a mediator of Alzheimer's disease (AD) (Head et al., 2009). Drp1 can be also activated by sumoylation (Wasiak et al., 2007). Thus, SUMO-1 and its conjugating enzyme Ubc9 induce mitochondrial fission by stabilizing Drp1 (Harder et al., 2004). In contrast, the sentrin/SUMO-specific protease SENP5 reduces Drp1 levels, which reduces mitochondrial fission (Zunino et al., 2009). In addition, it has been proposed that MARCH V, a mitochondrial E3 ubiquitin ligase, participates in the translocation of Drp1 to mitochondria independently of changes in stability (Karbowski et al., 2007). Phosphorylation occurs on Ser616 and Ser637 (in reference to the human Drp1 sequence). Phosphorylation of Ser616 by cyclin-dependent kinase 1 (Cdk1/cyclin B) causes Drp1 recruitment to the mitochondria (Taguchi et al., 2007). Phosphorylation of Ser637 is catalyzed by protein kinase A (PKA), Calmodulin-dependent kinase, and Pim1 (Chang and Blackstone, 2007; Cribbs and Strack, 2007), and it inhibits Drp1 function. Dephosphorylation of the Ser637 residue by the protein phosphatase calcineurin recruits Drp1 to the mitochondria and promotes fission (Cribbs and Strack, 2007; Cereghetti et al., 2008).

Drp1 ablation in mice causes embryonic lethality. Drp1 knockout embryos present alterations in liver and heart development, increased apoptosis within the deep neural cortex, and deficient synapse formation (Ishihara et al., 2009). In addition, Drp1 loss-of-function delays cytochrome c release during apoptosis, thereby suggesting that this release is linked to mitochondrial fission (Ishihara et al., 2009).

The recruitment of the Drp1 ortholog in yeast (Dnm1) from the cytosol to the outer mitochondrial membrane occurs through association with the protein Fis1, resulting in the formation of a fission complex (Mozdy et al., 2000; Legesse-Miller et al., 2003; Yoon et al., 2003; Karren et al., 2005). Mammalian Fis1 is a small ubiquitous 17.2-kDa protein found throughout the mitochondrial network. Fis1 is anchored into the outer mitochondrial membrane via its COOH terminal part, which contains an alpha-helix, a transmembrane domain, and a COOH-terminal tail exposed to the inter-membrane space. The NH2-terminal part of the protein contains four distinct regions with five alpha-helices (Suzuki et al., 2003; Dohm et al., 2004); the first alpha-helix of Fis1 has been reported to be critical for its oligomerization and fission activity (Jofuku et al., 2005). The subsequent four alpha-helices make up two tetratrico-peptide repeat peptides, which are not required for Fis1 oligomerization but they participate in the protein-protein interactions required for fission (Jofuku et al., 2005). Overexpression of Fis1 causes mitochondrial fragmentation, whereas knockdown of this protein results in the formation of a highly fused mitochondrial network, thereby indicating that Fis1 activates mitochondrial fission (Mozdy et al., 2000; Yoon et al., 2003; Stojanovski et al., 2004; Karren et al., 2005). However, the observations that Drp1 can still be recruited to the mitochondrial outer membrane following knockdown of Fis1 (Lee et al., 2004; Wasiak et al., 2007) suggest that other proteins also participate in mammalian mitochondrial fission.

Mff (Gandre-Babbe and van der Bliek, 2008; Otera et al., 2010) is anchored to the outer mitochondrial membrane and it can recruit Drp1 independently of Fis1 (Otera et al., 2010). Absent in yeast, Mff represents a more recent acquisition of the mitochondrial fission machinery that is active in mammalian cells. Mff localizes in discrete sites on mitochondria, and its overexpression causes recruitment of Drp1 to mitochondria and mitochondrial fragmentation. Conversely, Mff deficiency leads to reduced Drp1 at the mitochondrial and to mitochondrial elongation (Otera et al., 2010).

MiD49 and MiD51 proteins share 65% identity and are anchored to the mitochondrial outer membrane through their N-terminal end (Simpson et al., 2000; Palmer et al., 2011). Like Drp1, MiD49/51 form puncta and rings around mitochondria. MiD49/51 recruit Drp1 to the mitochondria, whereas their loss-of-function reduces mitochondrial Drp1 association. Co-immunoprecipitation experiments have established that MiD49 interacts with Drp1. MiD49 and MiD51 can act independently of both Mff and Fis 1 (Loson et al., 2013).

Located in the outer mitochondrial membrane, ganglioside-induced differentiation-associated protein 1 (GDAP1) has been proposed to participate in mitochondrial fission (Cassereau et al., 2011). GDAP1 gain-of-function causes mitochondrial fragmentation, while its deficiency results in mitochondrial elongation (Niemann et al., 2005). Genetic manipulation of the *Drosophila* ortholog Gdap1 also leads to changes in the size, morphology and distribution of mitochondria and in neuronal and muscular degeneration (Lopez Del Amo et al., 2015).

## Interaction of the endoplasmic reticulum and mitochondria: role of proteins involved in mitochondrial dynamics

**Figure 1:**
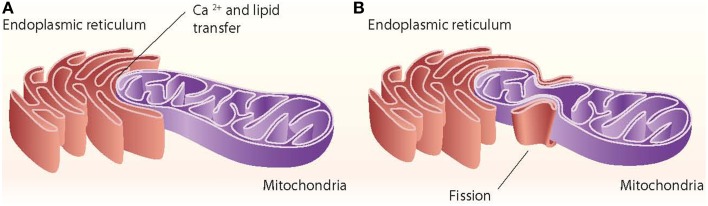
**ER–mitochondria contact sites**. The ER interacts with mitochondria through close contacts that permit the transfer of calcium from the ER to mitochondria and that permit the exchange of lipids between those two organelles **(A)**. The ER also interacts with mitochondria through mitochondrial fission sites **(B)**.

Close appositions between the endoplasmic reticulum (ER) and mitochondria or mitochondria-ER contact sites have been observed by electron microscopy in fixed samples of several cell types and are unexplainable by fixation artifacts. These regions represent the sites of phospholipid exchange between the two organelles. The close contacts through which the ER communicates with mitochondria have been biochemically purified and are referred to as the mitochondria-associated ER membrane (MAM) (Vance, 1990). The interaction between mitochondria and the ER is crucial in mediating organelle-organelle signals, such as metabolic stress or cell death cues (Figure 1).

**Figure 2:**
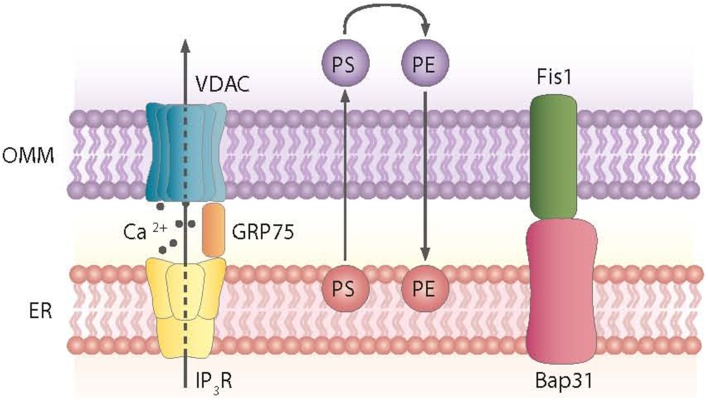
**Proteins and processes involved in the ER-mitochondria contact sites**. Contact sites involve, among others, the exchange of phosphatidylserine (PS) and phosphatidylethanolamine (PE) between the ER and mitochondria and the formation of complexes between the ER and mitochondrial proteins, as shown.

Mitochondria tethering to the ER via the MAM is a dynamic process. These ER-contiguous membranes contain multiple phospholipid- and glycosphingolipid-synthesizing enzymes, including long-chain fatty acid-CoA ligase type 4 (FACL4) and phosphatidylserine synthase-1, and they support the direct transfer of lipids between the ER and mitochondria (Stone and Vance, 2000; Cardenas et al., 2010). In addition to supporting lipid transfer, MAMs also exchange Ca^2+^ ions, which regulate processes such as ER chaperone-assisted folding of newly synthesized proteins and enzymes with dehydrogenase activity (Berridge, 2002). In this regard, the truncated variant of the sarcoendoplasmic reticulum Ca^2+^-ATPase 1 (SERCA1T) is induced during ER stress, promotes the transfer of calcium from the ER to mitochondria, and induces apoptosis (Chami et al., 2008). Nearly 30 proteins that play relevant roles in organelle homeostasis are found in this compartment. Furthermore, proteomic studies of mouse brain MAM fractions have identified about 1000 proteins localized in MAMs (Poston et al., 2013). Chaperones, ion channels, proteins of mitochondrial dynamics, and metabolic enzymes are among the molecules detected in MAMs. Thus, the chaperone GRP-75 links the outer mitochondrial membrane protein VDAC with the N-terminus of the inositol 1,4,5-trisphosphate receptor (IP3R), and GRP-75 deficiency reduces calcium transfer from the ER to mitochondria (Szabadkai et al., 2006) (Figure 2). Another MAM-localized protein is the Sigma-1 receptor chaperone. Sigma-1 binds GRP-78 and its overexpression counteracts the ER stress response and prevents apoptosis mediated by the release of ER calcium (Hayashi and Su, 2007). PACS2, another protein localized in MAMs, is involved in the ER-mitochondria interaction. PACS2 silencing causes mitochondria and ER fragmentation and also ER stress characterized by enhanced GRP-78 expression and reduced apoptosis (Simmen et al., 2005). The ER protein Nogo also alters the ER-mitochondria unit (Sutendra et al., 2011).

**Figure 3:**
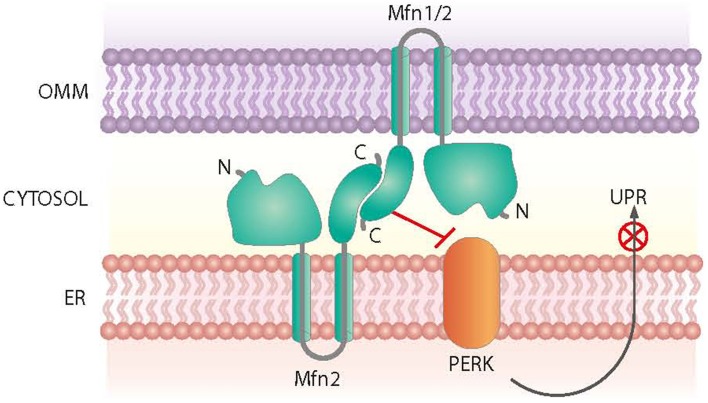
**Roles of Mfn2 on ER-mitochondria contact sites**. Mfn2 participates in the tethering of mitochondria and the ER at contact sites. In addition, Mfn2 negatively regulates PERK, a protein kinase involved in the Unfolded Protein Response (UPR) and activated upon ER stress.

Mfn2 was initially identified as a mitochondria fusion protein. However, it also plays a critical role in maintaining ER morphology by tethering mitochondria and the ER (de Brito and Scorrano, 2008) (Figure 3). *Mfn2* null mouse embryonic fibroblasts (MEFs) show a fragmented ER network, and the contact regions between mitochondria and the ER are significantly reduced (de Brito and Scorrano, 2008). In keeping with this view, Mfn2 loss-of-function in cells or in tissues such as muscle, liver or hypothalamus causes ER stress (Sebastian et al., 2012; Munoz et al., 2013; Schneeberger et al., 2013). Moreover, Mfn2 deficiency causes chronic activation of PERK, a process that plays a relevant role in the alterations detected under these conditions (Figure 3). Thus, PERK deficiency in Mfn2 null MEFs reduces ROS production, normalizes mitochondrial calcium, and improves mitochondrial morphology (Munoz et al., 2013).

Other proteins involved in mitochondrial dynamics, such as Drp1 and Fis1, are also associated with ER-mitochondria contact sites (Pitts et al., 1999; Iwasawa et al., 2011). In mammalian cells, the ER tubules contact the mitochondrial membrane at constriction sites marked by Drp1 and its receptor the Mff protein (Friedman et al., 2011). In fact, Drp1 and Mff are markers for ER-mitochondria contact sites. The ER-mitochondria contact is not disrupted by Drp1 or Mff depletion, thereby suggesting that such interactions are independent of division machinery recruitment (Friedman et al., 2011). Contact with the ER is therefore a feature of mitochondrial fission sites (Figure 1). This contact is also maintained after mitochondrial fission.

In all, alterations in the expression of Mfn2 lead to the disruption of ER-mitochondria communications and the ER plays a key role in the localization of mitochondrial fission sites.

## Mutations in genes involved in mitochondrial dynamics cause neurodegenerative disorders

In the nervous system, mitochondria are essential for energy production, calcium regulation, maintenance of plasma membrane potential, protein folding by chaperones, axonal and dendritic transport, and release and re-uptake of neurotransmitters at synapses. In the last years, mitochondrial dynamics has been reported to participate in the pathophysiology of neuronal disorders. In this respect, mutations in genes involved in mitochondrial dynamics have been shown to cause neuronal disorders.

### Autosomal dominant optic atrophy

Autosomal dominant optic atrophy (ADOA) has an estimated prevalence ranging from 1:12,000 to 1:50,000 and it is the most common form of inherited optic neuropathy. This disease is characterized by visual impairment in early childhood with moderate to severe loss of visual acuity, temporal optic disc pallor, abnormalities of color vision, and caecocentral visual field scotoma (Hoyt, 1980; Votruba et al., 1998; Johnston et al., 1999). Histopathological analysis suggests that the fundamental pathology of ADOA is a primary degeneration of retinal ganglion cells, followed by increasing atrophy of the optic nerve (Johnston et al., 1979; Kjer et al., 1983).

Most families with ADOA show pathogenic mutations in the *OPA1* gene (Olichon et al., 2006). More than 200 OPA1 gene mutations have been identified—these basically family-specific (Ferré et al., 2005). OPA1 mutations involve mainly substitutions, but also deletions and insertions (Delettre et al., 2001; Olichon et al., 2006). Near 50% of the mutations cause truncation of the OPA1 protein, and most of the mutations are detected in the GTPase domain and are thus likely to eliminate mitochondrial fusion. The pathogenesis of ADOA occurs, in most cases, as a result of haploinsufficiency (loss-of-function, Delettre et al., 2000). In this regard, OPA1 deficiency in mice induced by in-frame deletion of 27 amino acid residues in the GTPase domain or non-sense mutations causes the degeneration of retinal ganglion cells and disorganized mitochondrial cristae of optic nerve axons (Alavi et al., 2007; Davies et al., 2007). In addition to haploinsufficiency, ADOA can also develop as a result of a dominant negative mechanism (Delettre et al., 2000; Pesch et al., 2001; Baris et al., 2003; Kim et al., 2005).

Some human OPA1 mutations cause a specific form of disease, characterized by myopathy and progressive external ophtalmoplegia (Hudson et al., 2008), and has been named “OPA1 plus syndrome.” This syndrome is also characterized by reduced mitochondrial DNA copy number. In keeping with this, patients with certain OPA1 mutations show multiple deletions in mitochondrial DNA in skeletal muscle (Amati-Bonneau et al., 2008; Hudson et al., 2008), and mice carrying the Opa1(delTTAG) mutation (found in 30% of all human patients with ADOA) show a multi-systemic phenotype (Sarzi et al., 2012).

### Mfn2 mutations: Charcot-Marie-Tooth type 2A

Charcot-Marie-Tooth (CMT) disease is clinically characterized by weakness and distal muscle atrophy, predominantly of the lower extremities, and by sensory loss. CMT affects approximately 1 in 2500 individuals, thus making this condition one of the most common hereditary diseases and the most common hereditary neuropathy (Skre, 1974).

CMT2 is characterized by chronic axonal degeneration and regeneration, leading to steady loss of nerve fibers with normal or slightly reduced motor nerve conduction velocities (≥38 m/s) (Dyck and Lambert, 1968). Mutations in MFN2 cause 20% of CMT2 cases (Lawson et al., 2005), and this the most prevalent axonal form of CMT. CMT2 cases show high variability in clinical symptoms. Furthermore, MFN2 mutations have been detected in CMT2 families and are associated with additional features such as spasticity (Zhu et al., 2005) and atrophy (Verhoeven et al., 2006).

Most of the MFN2 mutations found in CMT2A patients are missense (Zuchner et al., 2004; Kijima et al., 2005; Lawson et al., 2005; Zhu et al., 2005; Chung et al., 2006; Engelfried et al., 2006; Verhoeven et al., 2006). More than 50% are detected in the GTPase domain but they do not affect GTP binding (Baloh et al., 2007). In addition, MFN2 mutations have been detected in the following sites: in the NH2-terminal region; near or at the Ras-binding domain; in the vicinity of or at the HR1 region; and in the COOH terminus, specifically in the HR2 region, facing the cytoplasmic site.

The mutations of MFN2 responsible for CMT2A show autosomal dominant inheritance; consequently, they may show haploinsufficiency or a dominant gain-of-function. In keeping with this view, the overexpression of some mutant forms of MFN2 induces the aggregation of mitochondria in cultured rat dorsal root ganglion neurons and in MEFs, indicating the promotion of gain-of-function (Baloh et al., 2007; Detmer and Chan, 2007). The defective mitochondrial fusion activity of some Mfn2 mutants is rescued by Mfn1 gain-of-function, which is in keeping with the observation that Mfn1 physically associates with wild-type Mfn2 and mutant forms of CMT2A (Loiseau et al., 2007; Amiott et al., 2008).

Transgenic mice have been generated to express a mutant form of MFN2 with a presumed gain-of-function (T105M) specifically in motor neurons. These mice show a phenotype consistent with the clinical symptoms detected in CMT2A, and provide a system to determine the function of mitochondria in the axons of motor neurons (Detmer et al., 2008).

GDAP1 mutations are associated with type 4A CMT (CMT4A), the most frequently detected recessive form of CMT (Baxter et al., 2002; Cuesta et al., 2002). CMT4 is classically defined as a demyelinating form of the disease and it is associated with segmental de- and re-myelination, in contrast to CMT2, which shows axonal degeneration without demyelination (Berger et al., 2002; Suter and Scherer, 2003). However, analysis of the nerve conduction rates of subjects with GDAP1 mutations indicates that while some mutations show low nerve conduction rates (Baxter et al., 2002; Ammar et al., 2003; Senderek et al., 2003), others are characterized by normal rates (Nelis et al., 2002; Ammar et al., 2003; Boerkoel et al., 2003; De Sandre-Giovannoli et al., 2003; Sevilla et al., 2003). These data demonstrate that some of the GDAP1 mutations cause axonal loss rather than demyelinization.

## Mitochondrial dynamics and prevalent neurodegenerative diseases

Both fusion and fission mechanisms participate in the mitochondrial life cycle and any disruption of their balance can alter the steady-state distribution of mitochondria. Specifically in neurons, the mitochondrial fusion/fission machinery is intimately and critically involved in the formation of synapses and dendritic spines. Thus, alterations in mitochondrial dynamics prevent these organelles from distributing to synapses, thus leading to a loss of mitochondria from dendritic spines and, consequently, to a reduction of synapse formation (Li et al., 2004). The mitochondrial dynamics proteins that control the distribution of mitochondria in dendrites also regulate the density and plasticity of synapses (Li et al., 2004). Thus, dominant-negative Drp1 or OPA1 overexpression (both of which reduce dendritic mitochondria) causes a decreased density of spines and synapses (Li et al., 2004). Consistent with this view, Drp1 ablation in mice shows developmental abnormalities, particularly in the forebrain (Ishihara et al., 2009). Given these considerations, a strong association between the expression/activity of proteins involved in mitochondrial dynamics and neurodegenerative diseases can be expected.

In this regard, brains from AD patients show fragmented and perinuclear mitochondria (Cho et al., 2009; Wang et al., 2009a), paralleled by increased expression of Fis1 and decreased expression of the fusion proteins Mfn1, Mfn2, and OPA1 (Wang et al., 2009a; Reddy et al., 2011). These data suggest that abnormal mitochondrial dynamics in neurons of AD patients may participate in the pathogenesis of the disease. In line with these observations, brains from AD patients show altered lipid metabolism (Schon and Area-Gomez, 2010), aberrant calcium homeostasis (Supnet and Bezprozvanny, 2010), enhanced unfolded protein response (UPR) (Hoozemans et al., 2005), and defects in energy metabolism (Ferreira et al., 2010). As to the factors responsible for mitochondrial dysfunction, overexpression of amyloid precursor protein (APP) or exposure to amyloid beta peptide (Abeta) induces mitochondrial fragmentation and abnormal distribution (Rui et al., 2006; Wang et al., 2008). Furthermore, exposure of neurons to oligomerized Abeta leads to S-nitrosylation of Drp1, causing mitochondrial fragmentation (Cho et al., 2009). Upregulated MAM function at the ER-mitochondrial interface and increased cross-talk between these two organelles has been reported in presenilin-mutant cells, and it may participate in the pathogenesis of AD (Area-Gomez et al., 2012).

Complex I activity is reduced in the substantia nigra of subjects with Parkinson's disease (PD), and various complex inhibitors (MPP^+^, rotenone, and other pesticides) cause neuropathological changes similar to those observed in PD (Schapira et al., 2006). In this regard, rotenone and 6-hydroxydopamine have been shown to induce Drp1-dependent mitochondrial fragmentation in neuronal cells (Barsoum et al., 2006; Gomez-Lazaro et al., 2008). Mutations in Parkin and outer membrane kinase PTEN-induced putative kinase-1 (PINK1) have been reported in familial PD, and these proteins participate in a major mitochondrial quality control pathway (Pickrell and Youle, 2015). When the mitochondrial membrane potential decreases, PINK1 recruits Parkin to the mitochondria (Narendra et al., 2010). Parkin next ubiquitinates PINK1, thus trigering mitophagy. Parkin also ubiquitinates other targets such as Mfn1, Mfn2, Fis1, and Drp1, causing their proteasomal degradation (Tanaka et al., 2010; Ziviani et al., 2010; Chan et al., 2011; Wang et al., 2011). In agreement, loss of function of PINK1 or Parkin causes mitochondrial fragmentation (Exner et al., 2007; Lutz et al., 2009; Sandebring et al., 2009), and mammalian fibroblasts carrying PINK1 mutations from PD patients also exhibit mitochondrial fragmentation (Grunewald et al., 2009).

Huntington's disease (HD) is caused by an expansion of a CAG trinucleotide sequence that encodes a polyglutamine tract in the huntingtin protein. Mitochondrial dysfunction is also associated with the pathogenesis of HD (Brouillet et al., 1995; Tabrizi et al., 1999; Panov et al., 2002; Schapira et al., 2014). Thus, the striatum and frontal cortex of HD patients show an increased abundance of Fis1 and Drp1 (Costa et al., 2010) and decreased levels of Mfn1, Mfn2, and OPA1 (Shirendeb et al., 2011). This pattern of changes may lead to mitochondrial fragmentation in these subjects. As to the mechanisms responsible for mitochondrial fragmentation, overexpression of mutant huntingtin causes mitochondrial fragmentation and blockade of fragmentation prevents the cytotoxic effects of mutant huntingtin (Wang et al., 2009b). Furthermore, mutant huntingtin binds to Drp1 and enhances its GTPase activity (Song et al., 2011). In all, these studies support the notion that alterations in mitochondrial dynamics are involved in the pathogenesis of HD.

## Hypothalamic neuronal circuits and mitochondrial function

The maintenance of energy homeostasis is fundamental to sustain life. In mammals, the central nervous system (CNS) plays a critical role in the regulation of appetite, energy expenditure, and metabolism through multiple and distributed neuronal circuits. The hypothalamus, which is made up of distinct nuclei, including the arcuate nucleus (ARC), the paraventricular nucleus, the lateral area, the dorsomedial nucleus and the ventromedial nucleus, is arguably the most important CNS region in metabolic control (Schneeberger et al., 2014). Extensive experimental evidence indicates that ARC is a key area in the neural hierarchy that regulates this biological process. The ARC holds at least two subsets of neurons—with opposite functions and reciprocally regulated—involved in systemic energy balance control. One set co-expresses orexigenic neuropeptides Agouti-related protein (AgRP) and Neuropeptide Y (NPY), while the other co-expresses anorexigenic neuropeptides cocaine- and amphetamine-regulated transcript (CART) and α-melanocyte-stimulating hormone (α-MSH, a product of proopiomelanocortin (POMC) processing) (Schneeberger et al., 2014).

### The melanocortin system

POMC and AgRP neurons, together with downstream neurons expressing melanocortin receptors (MCR) 3 and 4, form the melanocortin system, a crucial neuronal circuit that senses and responds to fluctuations in central and circulating factors that inform about the nutritional status of the organism. Indeed, the activity of these two subsets of neurons is influenced by local (NPY, serotonin, GABA, etc.) and peripheral (leptin, insulin, ghrelin) signals, as well as by nutrients such as glucose and free fatty acids. Numerous pharmacological and genetic studies indicate that the divergent physiological roles of these two populations of neurons are largely the consequence of the release and action of α-MSH and AgRP neuropeptides. α-MSH is an endogenous agonist of MCR3 and 4, thus providing an anorexigenic tone by suppressing appetite and increasing thermogenesis (Poggioli et al., 1986; Wirth et al., 2001). In contrast, AgRP is an antagonist of these receptors and therefore counteracts the effects of α-MSH signaling on food intake and body weight (Ollmann et al., 1997). In addition, the orexigenic actions of AgRP neurons are also mediated by the release of NPY, which binds to specific receptors, and by direct GABAergic synapsis onto POMC neurons (Cowley et al., 2001).

In summary, current evidence indicates that a local ARC circuit constituted by “first order” POMC and AgRP neurons plays a critical role in sensing, integrating, and responding to humoral signals that report on energy status. These neurons engage downstream “second order” multi-level neurocircuits that will produce precise effector responses. The global integration of these signals culminates in the generation of coordinated behavioral, autonomic, and neuroendocrine responses to regulate appetite, energy expenditure, and body weight (Schneeberger et al., 2014).

### Leptin and ghrelin: key hormones regulating energy homeostasis

Leptin and ghrelin are prototypical examples of metabolic signals of energy surfeit and deficit respectively, and POMC and AgRP neurons are direct targets of both hormones (Cheung et al., 1997; Elias et al., 1999; Willesen et al., 1999; Cowley et al., 2001). Leptin exerts its anorexigenic effects by increasing *Pomc* expression and processing into α-MSH (Schwartz et al., 1997; Thornton et al., 1997; Mizuno et al., 1998), and by inhibiting both *Npy* and *AgRP* transcription (Stephens et al., 1995; Schwartz et al., 1996; Mizuno and Mobbs, 1999). Leptin also increases the electrical activity of POMC neurons, thereby leading to α-MSH release (Cowley et al., 2001; Claret et al., 2007; Hill et al., 2008; Al-Qassab et al., 2009). In addition to its agonism/antagonism effects on MCRs, leptin attenuates the inhibitory GABAergic tone of AgRP neurons onto POMC neurons (Cowley et al., 2001). Similarly, recent data showed that leptin also acts on non-AgRP presynaptic GABAergic neurons by reducing the inhibitory input on POMC neurons (Vong et al., 2011). Overall, these effects result in reduced food intake and increased energy expenditure.

Under conditions of negative energy balance, circulating ghrelin levels are increased. Evidence indicates that the orexigenic effects of ghrelin are largely mediated by AgRP neurons (Chen et al., 2004b; Luquet et al., 2007; Wang et al., 2014). Consistently, ghrelin enhances *Npy* and *AgRP* transcript expression (Kamegai et al., 2001; Nakazato et al., 2001). The release of AgRP and NPY hyperpolarizes neighboring POMC neurons, thus reducing α-MSH release (Roseberry et al., 2004; Smith et al., 2007; Cyr et al., 2013). Furthermore, electrophysiology studies have shown that ghrelin activates AgRP neurons and increases the number of inhibitory synapses onto POMC neurons (Cowley et al., 2003; Yang et al., 2011; Atasoy et al., 2012). Collectively, these events lead to the simultaneous activation of AgRP neurons and inhibition of POMC neurons, thus enhancing the orexigenic output.

### Mitochondrial function in hypothalamic ARC neurons

Neurons are highly energy-demanding, and thus adequate mitochondrial performance is essential for the growth, survival, and function of these cells (Zhu et al., 2012). Mitochondrial ATP production and Ca^2+^ buffering/release are fundamental for a myriad of neuronal functions, including synapse assembly, action potential generation, and synaptic transmission, amongst others. Given their particular and intricate morphology, neurons require the distribution of mitochondria to distal areas in order to cover the high energy demands associated with a number of specialized functions. Thus, mitochondrial function and dynamics must be tightly regulated in order to temporally and spatially satisfy the bioenergetic needs of neurons.

Despite the paramount importance of the regulation of systemic energy balance and metabolism by mitochondria in hypothalamic neurons, few studies have addressed this issue. An important aspect to note is the diametrically different bioenergetic patterns of POMC and AgRP neurons, which are largely the consequence of their divergent physiological functions. Negative energy balance acutely activates AgRP neurons and promotes mitochondrial uncoupling activity associated with elevated mitochondrial density (Coppola et al., 2007; Dietrich et al., 2013). Under these conditions, ghrelin-induced β-oxidation constitutes the main energy supply to maintain AgRP neuron activity. This is achieved through an axis formed by the AMP-activated protein kinase (AMPK) and the muscle isoform of carnitine O-palmitoyltransferase 1 (CPT1-M) (Andrews et al., 2008; Lopez et al., 2008) which allows the uptake of long-chain fatty acyl CoAs by the mitochondria and the subsequent β-oxidation. A key component of this process is uncoupling protein 2 (UCP2), a mitochondrial protein that mediates proton leak, thereby limiting ATP and ROS production (Mailloux and Harper, 2011). Hypothalamic UCP2 expression is induced by fasting (Coppola et al., 2007), and ghrelin-driven feeding by AgRP requires UCP2 (Andrews et al., 2008). In line with these observations, fasting and ghrelin increase mitochondrial proliferation, synaptic plasticity, and electric activity of AgRP neurons in a UCP2-dependent manner (Coppola et al., 2007; Andrews et al., 2008). Under conditions of positive energy balance, when circulating leptin and glucose levels are high, POMC neurons increase their firing rate while AgRP neurons remain silent (Claret et al., 2007; Parton et al., 2007). Consistently, feeding conditions are associated with increased mitochondrial number in POMC neurons (Diano et al., 2011). The current model suggests that cellular glucose metabolism in POMC neurons generates a high proton gradient, concomitant with elevated ATP and ROS production. ATP would inactivate ATP-sensitive potassium (K_ATP_) channels and depolarize POMC neurons (Ibrahim et al., 2003). In addition, enhanced ROS levels would activate POMC neurons, further contributing to satiety (Diano et al., 2011). Mitochondrial UCP2 may be a key integrator of these processes, as its activity may limit ATP and/or ROS production and thereby modulate neuronal activity.

Collectively, these lines of evidence support the notion that mitochondrial processes and intermediates in ARC hypothalamic neurons play fundamental roles in sensing and integrating metabolic cues and also mediate neuronal function and associated behavior.

## Role of mitochondrial fusion proteins on hypothalamic ARC neurons

Recent research suggests that mitochondrial dynamics, in addition to constitute a key quality control mechanism, is also implicated in sensing nutrient fluctuations and promoting bioenergetic adaptations to meet the cellular metabolic needs. In general terms, physiological processes associated with enhanced energy demand and reduced energy supply (acute stress, starvation, etc.) are characterized by mitochondrial fusion and coupled respiration. In contrast, physiological situations associated with decreased energy demand and increased supply (high nutrient levels, obesity, type 2 diabetes) correlate with mitochondrial fission and decreased coupling (Liesa and Shirihai, 2013; Gao et al., 2014). Given that hypothalamic POMC and AgRP neurons are metabolic transceivers, it is reasonable to speculate that mitochondrial dynamics is an integral part of the molecular mechanisms by which these neurons sense the energy/nutritional milieu and mediate specific physiological actions to maintain energy homeostasis.

**Figure 4:**
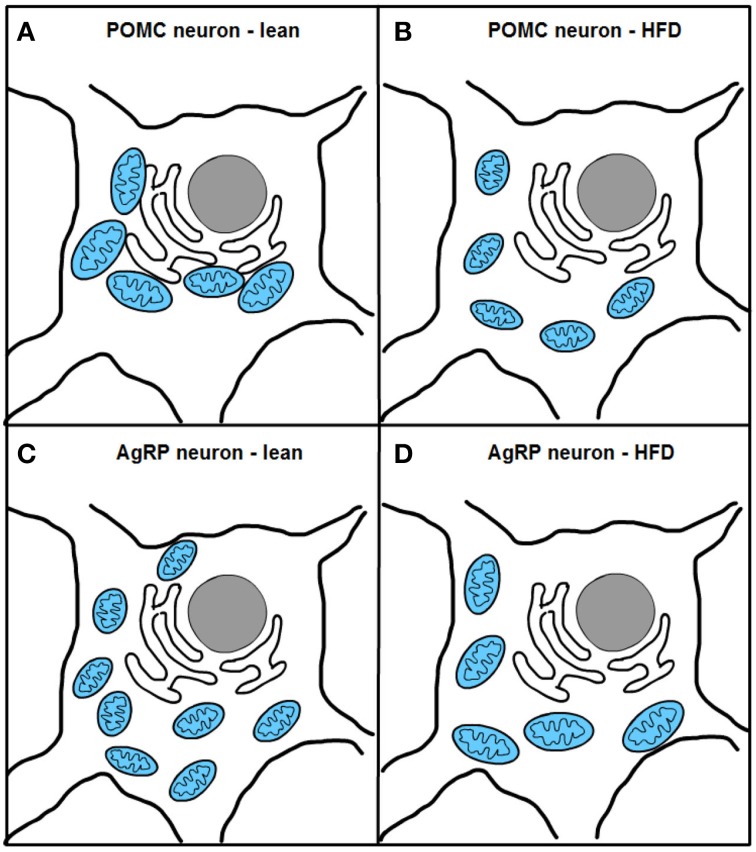
**Excess nutrient availability alters ER-mitochondria contacts and mitochondrial dynamics in POMC and AgRP neurons**. High-fat diet (HFD) administration increases mitochondrial fragmentation and impairs ER-mitochondria contacts in POMC neurons **(A,B)**. In contrast, AgRP neurons under HFD conditions show increased fusion and unaltered ER-mitochondria contacts **(C,D)**.

Two recent studies, one from our research group, have investigated mitochondrial dynamics and the role of fusion proteins Mfn1 and 2 in POMC and AgRP neurons upon energy balance control *in vivo* (Schneeberger et al., 2013). Electron microscopy studies under fasting conditions showed decreased mitochondria number but unaltered morphology in POMC neurons, while AgRP neurons exhibited elevated mitochondrial density and reduced size suggesting enhanced fission. High-fat diet (HFD) administration reduced mitochondria number but increased their size and elongation in AgRP neurons, in line with increased fusion (Dietrich et al., 2013). In contrast, POMC neuron mitochondrial elongation and network complexity was reduced, indicating elevated fission (Schneeberger et al., 2013) (Figure 4). Together, these results demonstrate specific patterns of mitochondrial dynamics in response to nutrient challenges in POMC and AgRP neurons, which is consistent with their divergent physiological functions and fuel usage.

**Figure 5:**
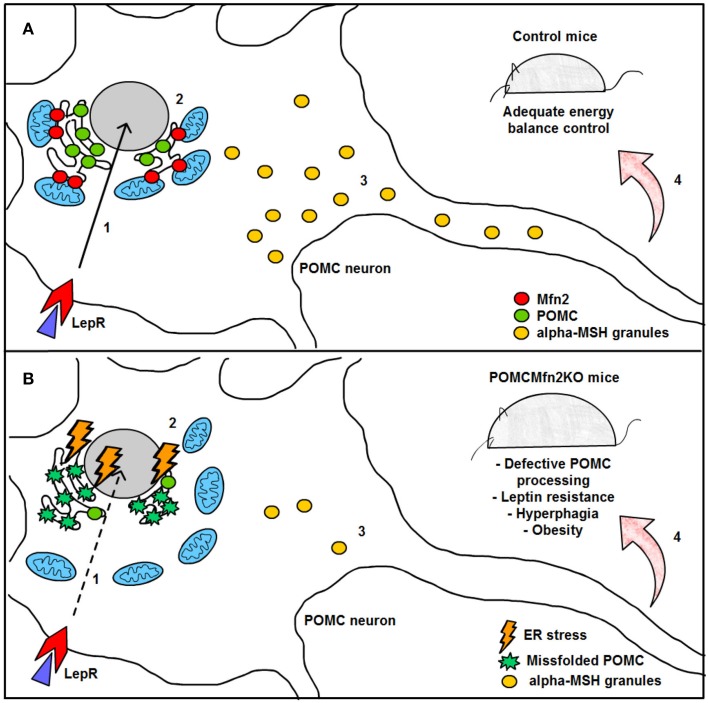
**Graphical summary of the consequences of Mfn2 loss in POMC neurons. (A)** Under normal physiological conditions, leptin signaling in POMC neurons is adequately transmitted, thereby enhancing POMC transcription in the nucleus (1) and subsequent synthesis in the ER (2). POMC precursor is sorted into secretory granules and processed into α-MSH (3). This neuropeptide is then released into target areas by POMC neuron axonal terminals thus mediating the anorexigenic effects of leptin (4). **(B)** Deletion of Mfn2 specifically in POMC neurons causes loss of ER-mitochondria contacts, thus leading to ER stress which interferes with proper POMC folding (2). Missfolded POMC precursor can not be adequately sorted or processed into α-MSH (3). As a consequence, leptin signaling (1) and leptin-mediated anorexigenic effects are blunted (4). LepR; leptin receptor; Mfn2: mitofusin 2; POMC: proopiomelanocortin; α.MSH: alpha- melanocyte stimulating hormone.

Remarkably, HFD feeding reduced the number of mitochondria-ER contacts in POMC neurons by ~50%, a defect that was not observed in the AgRP subpopulation (Figure 4). Recent reports have shown that Mfn2 is implicated in the tethering of mitochondria with ER (de Brito and Scorrano, 2008) and in the modulation of ER stress responses (Papanicolaou et al., 2012; Sebastian et al., 2012; Munoz et al., 2013). Indeed, under diet-induced obesity (DIO) conditions hypothalamic Mfn2 expression was reduced and its overexpression in the ARC was able to ameliorate the metabolic disturbances and reduce ER stress markers in the hypothalamus of DIO mice (Schneeberger et al., 2013). Consistent with these observations, conditional deletion of Mfn2 in POMC neurons resulted in a marked obesogenic phenotype as a consequence of loss of mitochondria-ER contacts and early development of ER stress-induced leptin resistance (Figure 5). In recent years, hypothalamic ER stress has emerged as a causal factor in the development of leptin resistance and obesity (Zhang et al., 2008; Ozcan et al., 2009) and this study established Mfn2 as a key molecular determinant connecting these processes. Interestingly, the number of mitochondria-ER junctions in POMC neurons may be a potential readout of leptin sensitivity (Schneeberger et al., 2013; Long et al., 2014). The specificity of this dramatic obese phenotype was demonstrated by the lack of energy balance abnormalities in mice lacking the homologous Mfn1 in the same population of neurons (Schneeberger et al., 2013).

Dietrich and associates (Dietrich et al., 2013) also used genetic conditional approaches to investigate the roles of Mfn1 and Mfn2 in AgRP neurons. The metabolic alterations observed in these mouse models were minimal under chow diet conditions (only females showed a slight metabolic improvement). However, a significant observation was that administration of HFD did not lead to the mitochondrial fusion in AgRP-specific knockout mice, suggesting that Mfn1- and Mfn2-mediated fusion was required for the bioenergetic adaptations of this population of neurons to positive energy balance (Schneeberger et al., 2013). This could be the consequence of reduced firing rate and increased number of silent AgRP neurons seen these dietary conditions.

Collectively, and in line with previous studies (Chen et al., 2007; Papanicolaou et al., 2011, 2012; Lee et al., 2012; Pham et al., 2012), these results reinforce the idea of non-overlapping physiological roles for mitofusin proteins and neuron-specific functions. For example, Mfn2 in POMC neurons is particularly involved in maintaining mitochondria-ER contacts and in the modulation of the cellular responses to ER stress, while in AgRP neurons mediates mitochondrial dynamics. It is interesting to note that in other neuronal types, but not in POMC and AgRP neurons, mitofusin proteins are essential for neuron survival and axon growth (Chen et al., 2007; Lee et al., 2012; Pham et al., 2012). The precise description of the basis of such divergent cell-specific biological functions will be of capital importance to understand the role of mitochondrial dynamics in hypothalamic neurons upon the regulation of energy balance and metabolism. These mechanisms will be also important to design potential targeted therapeutical approaches to counteract highly prevalent metabolic disorders such as obesity and type-2 diabetes.

## Future perspectives

A major current medical need is the efficient treatment of prevalent diseases such as obesity, type 2 diabetes, Alzheimer's disease, Parkinson's disease, or Huntington disease. In this respect, it is relevant to identify druggable proteins that participate in the pathophysiology of those disorders, and that permit to stop the progression of the disease or to ameliorate many of the alterations associated to them.

With the turning of the century we have learnt that alterations in genes relevant in mitochondrial dynamics are sufficient to develop diseases, leading to certain forms of CMT and ADOA. Furthermore, data obtained in the last years support the view that alterations in mitochondrial dynamics may be involved in the pathogenesis of neurodegenerative diseases, and in the hypothalamic dysfunctions leading to dysregulated energy balance. Ample evidence indicates that some neurodegenerative disorders are associated with altered metabolic and appetitive behaviors that may be explained by abnormal hypothalamic function. Although it is tempting to speculate that such perturbations are the consequence of defective mitochondrial dynamics, to date direct experimental evidence has not been provided. Certainly, there is a need to fully demonstrate the role of mitochondrial dynamics in the pathogenesis of metabolic and neurodegenerative diseases, and its potential link, by using more sophisticated mouse models and in humans. In spite of these difficulties, we hope that the thorough understanding of the mechanisms that control mitochondrial dynamics, and the interaction between mitochondrial and endoplasmic reticulum will permit to enter into a phase in which some of the mitochondrial dynamics proteins or regulators are amenable for pharmacological manipulation.

### Conflict of interest statement

The authors declare that the research was conducted in the absence of any commercial or financial relationships that could be construed as a potential conflict of interest.
